# Application of methanol and sweet potato vine hydrolysate as enhancers of citric acid production by *Aspergillus niger*

**DOI:** 10.1186/s40643-017-0166-4

**Published:** 2017-07-27

**Authors:** Daobing Yu, Yanke Shi, Qun Wang, Xin Zhang, Yuhua Zhao

**Affiliations:** 1 College of Forestry and Biotechnology, Zhejiang Agriculture and Forestry University, Lin’an, 311300 Zhejiang People’s Republic of China; 2 College of Life Sciences, Zhejiang University, Hangzhou, 310058 Zhejiang People’s Republic of China

**Keywords:** Citric acid, Methanol, Sweet potato vines hydrolysate, Synergistic effect, *Aspergillus niger*

## Abstract

**Background:**

Agricultural waste is as an alternative low-cost carbon source or beneficial additives which catch most people’s eyes. In addition, methanol and sweet potato vine hydrolysate (SVH) have been reported as the efficient enhancers of fermentation according to some reports. The objective of the present study was to confirm SVH as an efficient additive in CA production and explore the synergistic effects of methanol and SVH in fermentation reactions.

**Results:**

The optimal fermentation conditions resulted in a maximum citric acid concentration of 3.729 g/L. The final citric acid concentration under the optimized conditions was increased by 3.6-fold over the original conditions, 0.49-fold over the optimized conditions without methanol, and 1.8-fold over the optimized conditions in the absence of SVH. Kinetic analysis showed that* Q*_p_,* Y*_p/s_, and* Y*_x/s_ in the optimized systems were significantly improved compared with those obtained in the absence of methanol or SVH. Further, scanning electron microscopy (SEM) revealed that methanol stress promoted the formation of conidiophores, while SVH could neutralize the effect and prolong Aspergillus niger vegetative growth. Cell viability analysis also showed that SVH might eliminate the harmful effects of methanol and enhance cell membrane integrity.

**Conclusions:**

SVH was a superior additive for organic acid fermentation, and the combination of methanol and SVH displayed a significant synergistic effect. The research provides a preliminary theoretical basis for SVH practical application in the fermentation industry.

## Background

Citric acid (CA) is a versatile organic acid with a broad range of uses in the food, pharmaceutical, beverage, and cosmetic industries because of its mild sour flavor, water solubility, strong complexation, and high level of biological safety. Currently, the global production of CA has reached 1.7 million tons per year, produced almost entirely through fermentation, with an annual growth rate of 5% (Kana et al. 2012). The major method for manufacturing CA relies on the submerged fermentation of starch-based or sucrose-based feedstock by the fungus *Aspergillus niger*. Karaffa and Kubicek (2003) summarized the important role of glycolysis, the process of excretion and transport of CA, and the critical fermentation variables for CA accumulation. Finally, they proposed that the type and concentration of the carbon source is probably the most crucial parameter for successful citric acid production. A yield of 95 kg CA per 100 kg was even achieved of supplied sugar using best fermentation strains. However, high energy coupled with raw material costs has pushed CA production into an unprofitable market (Dhillon et al. 2011).

In the past few years, the use of agricultural waste as an alternative low-cost carbon source or beneficial additives has received considerable attention. For example, carob pod (Roukas 1998), kiwi fruit peel (Hang and Woodams 1998), pineapple waste (Kumar et al. 2003), jackfruit waste (Angumeenal and Venkappayya 2005), sugar cane bagasse (Ali and Haq 2005), orange peel (Rivas et al. 2008), and apple pomace (Dhillon et al. 2011; Ali et al. 2015) have all been utilized for CA production by *Aspergillus* sp. Sweet potato (*Ipomoea batatas*) is the world’s seventh most important food crop after rice, wheat, potatoes, corn, barley, and cassava (FAO 1997), with a production exceeding 105 million metric tons per year worldwide (Ishida et al. 2000). However, only the tuberous roots of sweet potato are used as food in most areas, whereas the vines are discarded as agricultural waste (Ishida et al. 2000), causing environmental pollution and wasting resources. Sweet potato vines contain nutritional and functionally valuable components and represent a considerable source of carbon, nitrogen, and energy (Ishida et al. 2000). However, there are few relevant reports on the effective application of sweet potato vines in bio-industry, apart from previous research confirming that the combination of molasses and SVH was good alternative carbon source in lipid fermentation (Shen et al. 2013, 2015).

A preliminary study revealed that CA production was markedly increased when 1% methanol and 10% SVH were added to basal medium under standard fermentation conditions. The objective of the present study was to confirm SVH as an efficient additive in CA production and explore the synergistic effects of methanol and SVH in fermentation reactions. To achieve the best fermentation results, the response surface methodology (RSM) was used to optimize key variables, such as temperature, pH, inoculum quantity, aeration condition, and fermentation period. In addition, the mechanism of methanol- and SVH-enhanced CA production was assessed by cultivation dynamics, SEM, and flow cytometry (FCM) of *A. niger* cells under different incubation conditions. The research will provide a preliminary theoretical basis for SVH practical application in the fermentation industry.

## Methods

### Microorganism and medium

The fungus *Aspergillus niger* sp. ZJUY previously isolated in our laboratory was used in the present study. The PDA medium for harvesting spores contained 200.0 g/L peeled potato, 20.0 g/L glucose, and 20.0 g/L agar (pH not adjusted). The CA-fermentation basal medium contained 50.0 g/L potato starch supplemented with 20 μL/L α-amylase (20,000 U/mL), 50.0 g/L glucose, 3.0 g/L NH_4_Cl, 0.2 g/L MgSO_4_·7H_2_O (pH 6.3). SVH was prepared as described in Zhan et al. (2013). A single-factor analytic approach was adopted to determine the optimal concentration of methanol and SVH. RSM was utilized to optimize the CA-fermentation conditions as described below.

### Cultivation

ZJUY was inoculated on PDA agar and cultured at 28 °C for 96 h. Spores were eluted with 20 mL 0.1% (v/v) Tween-80, of which approximately 15 mL was filtered through lens paper, transferred to a sterilized 50-mL flat-top centrifuge tube, and then separated by centrifugation (2700×*g*, 5 min). The supernatant was removed and the spores resuspended in 20 mL of 0.1% (v/v) Tween-80, and then centrifuged again. The resulting pellet was resuspended in 3 mL sterile water and diluted to 1 × 10^9^ CFU/mL in seed broth. Fermentation experiments were performed in a 250-mL Erlenmeyer flask containing 100 mL medium and cultured at 37 °C at 200 rpm for 4 days.

### Determination of citric acid concentration

Citric acid concentration was determined with an Agilent 1200 Series high-performance liquid chromatography (HPLC) instrument equipped with a UV/Vis detector and Eclipse Plus C18 column (250 × 4.6 mm × 5 μm; Agilent Technologies, Santa Clara, CA, USA). The processed broth was diluted 10-fold in the phosphate buffer (25 mM, pH 2.4), filtered through a 0.22-μm membrane, and injected into 2.0-mL autosampler vials. CA was separated with a mobile phase composed of methanol and phosphate buffer (25 mM, pH 2.4) at a 1:9 ratio (v/v) with a flow rate of 1.0 mL/min at 30 °C. The injection volume was 20 μL and we performed three replicates of each trial. CA quantitation was performed at the wavelength of maximum absorbance for each analyte (*λ* = 210 nm; A_210_) obtained from UV spectrophotometry spectra determination (Rodrigues et al. 2007). CA was identified by comparing its retention time with that of a standard substance, and its concentration was quantified using an external standard calibration, calculated by the following equations:

1 $$C = k \times F$$

2 $$F = 9 \times 10^{ - 4} \times {{S}} - 0.0010$$

where, *k* is the dilution ration (*k* = 10),* F* is the linear regression function (*R* ^2^ = 1.0000), *S* is the peak area of CA at A_210_, and *C* is the CA concentration (g/L).

### Determination of the optimal levels of fermentation factors

Factors previously identified to affect CA production were analyzed in single-factor experiments, including temperature (°C; 20, 25, 30, 35, 40, 45), pH (3.5, 4.0, 4.5, 5.0, 5.5, 6.0, 6.5, 7.0), inoculum quantity (%; 0.5, 1, 3, 5, 8, 10, 12, 15), rotational speed (rpm; 80, 100, 120, 140, 160, 180, 200, 200, 220), fermentation period (d; 3, 4, 5, 6, 7, 8, 9, 10), methanol content (%; 1, 2, 3, 4, 5, 6, 7, 8), and SVH concentration (%; 1, 3, 5, 8, 10, 12, 15, 20). All trials were performed in triplicate as described above.

### Factorial design and optimization study of CA production

#### Plackett–Burman design

For screening purposes, seven independent variables were screened in 12 combinations organized according to the Plackett–Burman design (Table 1). All experiments were performed in triplicate and the average CA concentration was treated as the response. The optimal level of each single-factor experiment became the low level, while the high level was 1.25 times the low level. The main effect of each variable was calculated as the difference between the average of measurements made at the high setting (+) and the average of measurements observed at the low setting (−) for that factor.

**Table 1:** Plackett–Burman design for 7 variables and 12 trials

Trial	Variable							CA (g/L)
	pH	Temperature (°C)	Inoculum quantity (%)	Rotational speed (rpm)	Fermentation period (d)	Methanol content (%)	SVH (%)	
1	6 (−1)	44 (+1)	15 (+1)	125 (+1)	5 (−1)	6 (−1)	10 (−1)	1.917
2	7.5 (+1)	44	12 (−1)	125	6.25 (+1)	7.5 (+1)	10	1.856
3	7.5	44	12	100 (−1)	5	7.5	10	1.755
4	7.5	35 (−1)	12	100	6.25	6	12.5 (+1)	2.072
5	7.5	35	15	125	5	7.5	12.5	2.050
6	6	35	12	125	5	7.5	12.5	2.000
7	6	35	12	100	5	6	10	1.784
8	6	44	12	125	6.25	6	12.5	2.166
9	7.5	35	15	125	6.25	6	10	2.105
10	6	44	15	100	6.25	7.5	12.5	2.057
11	6	35	15	100	6.25	7.5	10	2.396
12	7.5	44	15	100	5	6	12.5	2.004

The (−1) indicates the low level

(+1) indicates the high level

#### Central composite design

Fermentation factors affecting CA production were optimized with CCD in Design-Expert software, version 10.0, (Stat-Ease Inc., Minneapolis, MN, USA) using 50 experimental runs and 5 variables. Each factor was examined at five different levels: relatively low (−), low (−), basal (0), high (+), and relatively high (++) (Table 2). The CCD results were fitted to a second-order polynomial model as follows:

3 $$\begin{aligned} Y = \, & \beta_{0} + \beta_{1} A + \beta_{2} B + \beta_{3} C + \beta_{4} D \\ & + \beta_{5} E + \beta_{12} AB + \beta_{13} AC + \beta_{14} AD \\ & + \, \beta_{15} AE + \beta_{23} BC + \beta_{24} BD + \beta_{25} BE \\ & + \beta_{34} CD + \beta_{35} CE + \, \beta_{45} DE + \beta_{11} A^{2} \\ & + \beta_{22} B^{2} + \beta_{33} C^{2} + \beta_{44} D^{2} + \beta_{55} E^{2} \\ \end{aligned}$$

where, *Y* is the dependent variable (CA concentration), *A* is the initial pH, *B* is temperature (°C), *C* is inoculum quantity (%), *D* is fermentation period (d), *E* is SVH concentration (%), *β*_0_ is the regression coefficient at the center point, and *β* is the estimated coefficient for each term of the response surface model.

**Table 2:** Independent variables and levels of variation in CCD

Trial	Variable					CA (g/L)
	pH	Temperature (°C)	Inoculum quantity (%)	Fermentation period (*d*)	SVH (%)	
1	4 (−)	35 (+)	12 (−)	7 (+)	12 (+)	2.011
2	6 (+)	35	18 (+)	7	10 (−)	2.485
3	6	25 (−)	18	7	12	2.032
4	5 (0)	30 (0)	15 (0)	6 (0)	13.4 (++)	2.426
5	6	25	12	7	10	2.292
6	5	30	15	6	11 (0)	2.588
7	4	35	18	5 (−)	10	2.216
8	4	35	12	5	12	2.579
9	6	35	12	7	10	2.617
10	4	25	18	7	12	2.284
11	4	35	18	7	10	2.263
12	4	35	18	5	12	2.211
13	6	25	18	5	12	2.852
14	5	30	8 (–)	6	11	2.775
15	5	30	15	6	11	2.803
16	5	42 (++)	15	6	11	2.581
17	6	25	12	7	12	2.466
18	4	25	18	5	12	2.027
19	5	30	15	6	11	2.716
20	5	30	15	6	11	2.685
21	6	25	18	5	10	2.245
22	4	35	18	7	12	2.062
23	5	30	15	6	11	2.788
24	4	25	12	7	12	2.264
25	4	25	12	5	10	2.303
26	5	30	22 (++)	6	11	2.855
27	6	35	18	5	10	2.916
28	6	35	18	5	12	3.682
29	6	25	12	5	10	2.413
30	4	25	12	7	10	2.226
31	6	35	18	7	12	2.591
32	4	25	18	5	10	1.985
33	4	25	12	5	12	2.148
34	6	25	12	5	12	2.583
35	6	35	12	7	12	2.383
36	5	30	15	8.4 (++)	11	2.286
37	2.6 (–)	30	15	6	11	1.981
38	5	18 (–)	15	6	11	2.132
39	6	35	12	5	10	2.851
40	7.4 (++)	30	15	6	11	2.171
41	5	30	15	6	11	2.741
42	5	30	15	6	8.6 (–)	2.733
43	5	30	15	3.6 (–)	11	2.078
44	5	30	15	6	11	2.407
45	5	30	15	6	11	2.593
46	4	25	18	7	10	2.541
47	4	35	12	7	10	2.628
48	4	35	12	5	10	2.619
49	6	35	12	5	12	2.882
50	6	25	18	7	10	1.953

Each factor was examined at 5 levels including (–), (−), (0), (+) and (++) which indicates the highest level

### Citrate synthase activity analysis

*Aspergillus niger* cell-free extracts were prepared as described by Kobayashiet al. (2013), and the protein concentrations determined with a Bradford Protein Assay Kit. CS activity was measured as previously described by Srere (1966) with some modifications. The reaction mixture contained 50 mM Tris–HCl (pH 8.0), 500 mM MgCl_2_, 5.0 mM 5,5′-dithiobis-(2-nitrobenzoic acid) (DTNB), 20 mM acetyl-CoA, 50 mM oxaloacetate, and crude enzyme solution in a total volume of 1.0 mL. One unit (U) was defined as the amount of enzyme catalyzing the liberation of 1 μmol of CoA-SH per min.

### Kinetic characterization of fermentation

Kinetic parameters of the CA-fermentation process were determined according to Pirt (1975). Kinetic parameters: *Q*_p_ = grams of CA produced/L/h, *Y*_p/s_ = grams of CA produced/gram of substrate consumed, *Y*_x/s_ = grams of cells/gram of substrate utilized, *Q*_s_ = grams of substrate consumed/L/h (Ali 2007).

### Observations of the mycelial morphology

Scanning electron microscope was used to observe changes in mycelial morphology under different cultural conditions. For this, the culture samples were centrifuged, washed, and the insoluble fractions subsequently resuspended in sterile water and fixed in 2.5% glutaraldehyde at 4 °C overnight. The fixed sample was washed separately with 1% osmium and PBS three times and then dehydrated with an ethanol gradient. The dehydrated samples were dried with critical point drier, and sputter-coated with gold before SEM analysis.

### Detection of cell viability

Filtered broth from 24-h cultures was centrifuged to collected spores, which were then diluted to approximately 10^6^ spores/mL. The spores were subsequently fixed with 0.2% oxymethylene, washed twice with PBS buffer, and then stained with 30 μg/mL propidium iodide (PI; Beyotime, China) at room temperature for 30 min. The stained spheroblasts were pelleted by centrifuging for 2 min at 16,000×*g* at 4 °C, washed, and then resuspended in 500 μL PBS buffer. Flow cytometry (FCM) coupled with PI was used to monitor *A. niger* membrane integrity and sorted at a rate of approximately 500 events per second, with 30,000 total events detected in each run. Flow cytometry was carried out using a BD FACSCalibur instrument (Becton–Dickinson, USA) fitted with a 15 mW argon ion laser for excitation (488 nm), while monitoring with three different emission channels (530/30, 585/42, and 670 nm Lp). BD CellQuest Pro software (Becton–Dickinson, USA) was used for instrument control, data acquisition, and data analysis.

## Results

### Single-factor optimization of fermentation

**Fig. 1:**
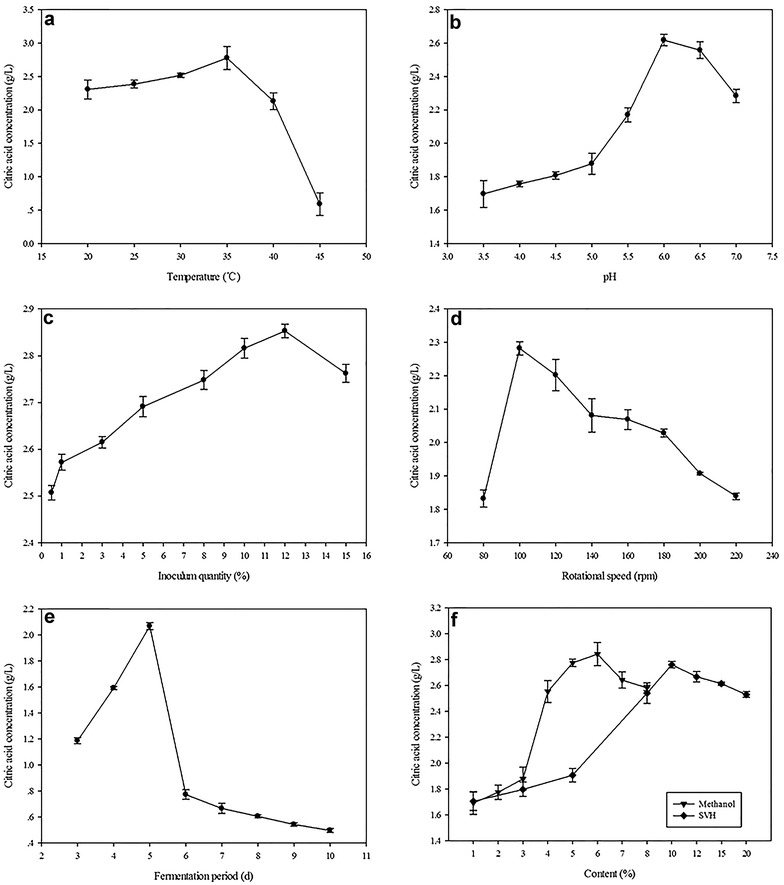
Single-factor optimization of fermentation condition: **a**–**e** effects of temperature, pH, inoculum quantity, rotational speed, fermentation period, and **f** the content of methanol and SVH on citric acid concentrations, respectively

Single-factor optimization was used to confirm the effects of temperature, pH, inoculum quantity, rotational speed, fermentation period, methanol content, and SVH concentration on CA production (Fig. 1). Notably, temperature showed a significant effect on CA concentration according to normal distribution with minor variation. The maximum CA concentration was observed at 35 °C (Fig. 1a), which was significantly decreased when the fermentation temperature was higher than 45 °C or lower than 20 °C. This phenomenon was correlated with biomass production (data not shown). Similar findings were reported by Karthikeyan and Sivakumar (2010).

Rault et al. (2009) specified the effects of fermentation pH on the physiological-state dynamics of *Lactobacillus bulgaricus* CFL1. The cells maintained a more vigorous physiological state when pH was controlled at 5, with higher viability and steady acidification activity, but these characteristics fluctuated and declined at pH 6 during the fermentation process. Moreover, pH also played an important role in CA accumulation (Fig. 1b), which increased with initial pH from 3.5 to 6.0, and a highest value (2.618 g/L) was achieved at an initial pH of 6.0. However, the CA concentration decreased dramatically once the initial pH exceeded 7.0, consistent with results from Roukas (1998) on the effect of initial pH on CA production from carob pods by surface fermentation.

Analysis of various *A. niger* inoculation levels on CA fermentation revealed that the maximum CA concentration was obtained with an inoculation quantity of 12% (Fig. 1c), equivalent to a final spore concentration of 10^8^ CFU/mL, similar to previous findings (Vandenberghe et al. 2000). An optimal inoculum level is critical for CA accumulation because low inoculums may provide inadequate biomass and limit CA formation, whereas excessive levels could generate too much biomass to retain sufficient nutrients necessary for CA production (Sabu et al. 2006).

In the bioreactor, agitation replaced the rotational shaking, but these techniques have similar effects on CA production. We selected the small-scale flask-shaking fermentation method for analysis. Rotational speed is known to affect both the mobility of fermentation broths and the rate of oxygen transfer (Amanullah et al. 1998). The maximum specific growth rate of the culture in the exponential phase was closely related to the amount of dissolved oxygen. As shown in Fig. 1d, the peak of CA concentration appeared at 100 rpm and decreased when the rotation speed exceeded the optimal level, perhaps due to the excessive fungal growth.

Different fermentation periods were compared to determine the optimal incubation time. Notably, the highest CA concentration was obtained at 5 days of culture in our study. Further incubation resulted in a sharp decreased in CA production (Fig. 1e), in agreement with results from Karthikeyan and Sivakumar (2010). However, the optimal period varied from material to material. For instance, the best fermentation time for *A. niger* was 5 days when cassava bagasse was used as a substrate (Vandenberghe et al. 2000), 4 days with jack fruit carpel fiber (Angumeenal and Venkappayya 2005) and orange peel autohydrolysate (Rivas et al. 2008).

Methanol is known to boost CA production by *A. niger* (Navaratnam et al. 1998), likely by stimulating its excretion by increasing cell membrane permeability, which can reduce the mass transfer resistance of the membrane and strengthen the catalysis of the cell, without damaging intracellular organic structures or causing cell lysis. (Rivas et al. 2008). Moreover, 2-oxoglutarate dehydrogenase activity was low, whereas that of pyruvate carboxylase was high in the presence of methanol. Maddox et al. (1986) also observed strong correlations between CA production and the activities of these two enzymes. In the present study, the maximum threshold of CA concentration was obtained with 6% methanol (Fig. 1f).

Figure 1f depicts the effect of SVH addition on CA production, which showed a steady increase as SVH increased from 1 to 10%, and the highest concentration (2.762 g/L) was achieved with 10% SVH. However, the gradually increasing trend was reversed with SVH concentrations greater than 10%.

### Evaluation of significant factors affecting CA concentration

The main effect of each variable on CA concentration was estimated as the difference between the average CA concentrations at the high (+1) and low (−1) levels. As shown in Table 3, all variables had significant effects on CA production during the fermentation period with the exception of rotational speed (from 100 to 125 rpm) and methanol content (from 6 to 7.5%), whereas fermentation period affected CA accumulation the most, followed by inoculation quantity, temperature, and SVH (*p* < 0.05).

**Table 3:** Analysis of variance (ANOVA) for Plackett–Burman design

Source	Sum of squares	*df*	*F* value	*p* value Prob > *F*	
Model	0.34	10	600.73	0.0317	significant
A-initial pH	0.019	1	337.99	0.0346	
B-temperature	0.035	1	628.85	0.0254	
C-inoculation quantity	0.067	1	1187.6	0.0185	
E-fermentation period	0.11	1	1929.24	0.0145	
F-methanol content	3.63E−04	1	6.44	0.2389	
G-SVH	0.024	1	424.99	0.0309	

### Optimization of fermentation conditions by CCD

Each independent variable was investigated at five levels according to the CCD. Table 2 represents the design matrix of the coded variables together, according to its result on CA concentration. Best-fit models were determined by quadratic regression. The modified quadratic model was a highly significant (*p* < 0.001) representation of the actual relationships between the responses and significant variables. ANOVA was used to evaluate the significance of the coefficients of the modified quadratic model (Table 4), and a second-order polynomial function was fitted to the experimental results using Design-Expert 10.0 software as follows:

**Table 4:** ANOVA and coefficient values for CCD

Source	Sum of squares	*df*	*F* value	*p* value Prob > *F*	
Model	3.26	20	5.11	<0.0001	Significant
*A*-initial pH	0.5	1	15.49	0.0005	
*B*-fermentation temperature	0.52	1	16.32	0.0004	
*C*-inoculum quantity	0.047	1	1.47	0.2351	
*D*-fermentation period	0.11	1	3.57	0.0690	
*E*-SVH	0.02	1	0.62	0.4391	
AB	0.13	1	4.16	0.0506	
AC	0.018	1	0.57	0.4579	
AD	0.3	1	9.39	0.0047	
AE	0.15	1	4.72	0.0382	
BC	1.32E−04	1	4.13E−03	0.9492	
BD	0.093	1	2.9	0.0995	
BE	0.079	1	2.47	0.1270	
CD	2.20E−03	1	0.069	0.7952	
CE	0.036	1	1.13	0.2975	
DE	0.083	1	2.6	0.1177	
*A* ^2^	0.64	1	20.1	0.0001	
*B* ^2^	0.19	1	5.84	0.0222	
*C* ^2^	0.03	1	0.93	0.3434	
*D* ^2^	0.44	1	13.71	0.0009	
*E* ^2^	0.019	1	0.6	0.4461	
Residual	0.93	29			
Lack of fit	0.81	22	2.13	0.1544	Not significant
Pure error	0.12	7			
Cor total	4.19	49			

Coefficient of determination (*R* ^2^) = 0.7788, CV = 7.31%. The “Pred R-Squared” of 0.2324 was not as close to the “Adj R-Squared” of 0.6262 as one might normally expect. The “Adeq Precision” value of 10.000 indicated an adequate signal

4 $$\begin{aligned} Y = \, & 2.66 + 0.11{\kern 1pt} A + 0.11B - 0.033C - 0.051D \\ & - 0.021E + 0.064 \text {AB} + 0.024 \text{AC} - 0.097 \text {AD} \\ & + \,0.069 \text {AE} - 2.031 \times 10^{ - 3} \text {BC} - 0.054 \text {BD} \\ & - 0.050 \text {BE} + 8.281 \times 10^{ - 3} \text {CD} + 0.034 \text {CE} \\ & -\, 0.051 \text {DE} - 0.11A^{2} - 0.058B^{2} + 0.023C^{2} \\ & - 0.089D^{2} - 0.019E^{2} \\ \end{aligned}$$

Solving the model according to the data obtained from Table 2 revealed an optimal response under the following conditions: 18% inoculum quantity, 12% SVH, 7.5% methanol, initial pH of 6.0, 125 r/min rotational speed, and culturing at 35 °C for 5 days. These factors produced a final CA concentration of 3.673 g/L, which was very similar to the predicted condition of Trial 28 in the CCD experiment.

**Fig. 2:**
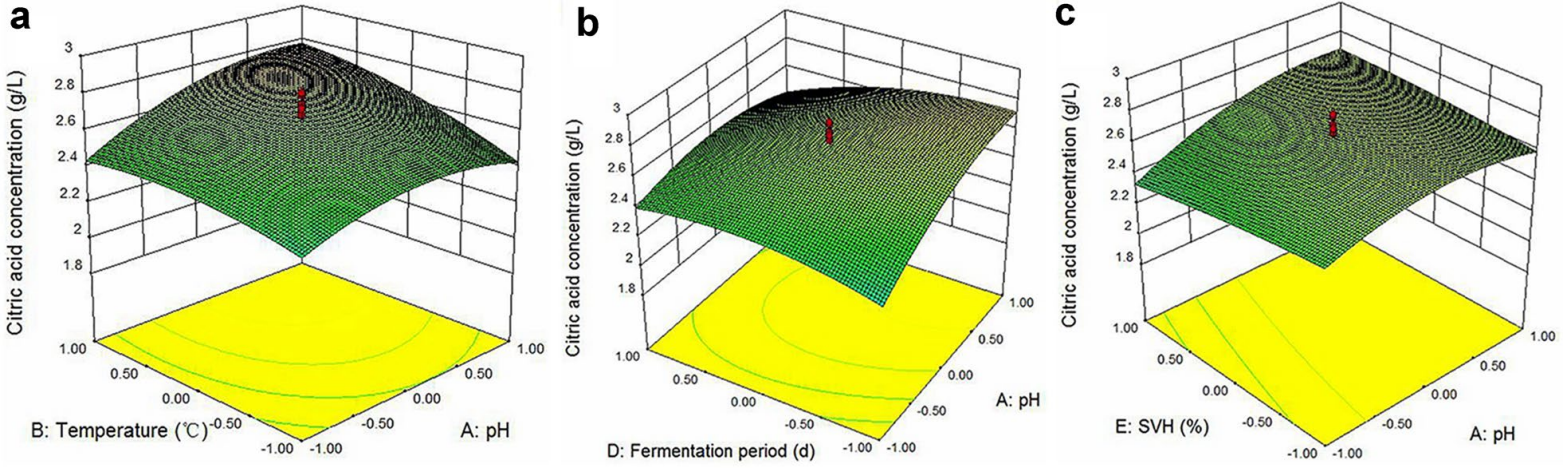
Response surfaces plots for *A. niger* sp. ZJUY showing the interactive effects of initial pH and fermentation temperature (**a**), initial pH and fermentation period (**b**), and initial pH and SVH (**c**) on citric acid concentration, respectively

Effects of the interaction of tested variables on the CA concentration were clearly represented in the three-dimensional response surface plots (Fig. 2). The results were analyzed by ANOVA which illustrated that the interactions of initial pH-fermentation temperature, initial pH-fermentation period, and initial pH-SVH significantly affected CA production (*p* < 0.05).

### Biochemical behaviors of *A. niger* in different fermentation systems

To confirm the model’s validity and explore the synergistic effects of adding both methanol and SVH to the CA-fermentation system, an experiment was performed under the predicted optimized conditions in the presence or absence of methanol and SVH. The original fermentation conditions were used as the control: initial pH 6.3, 37 °C fermentation temperature, 1% inoculum quantity, and 200 rpm rotational speed, with a 4-day fermentation period.

**Fig. 3:**
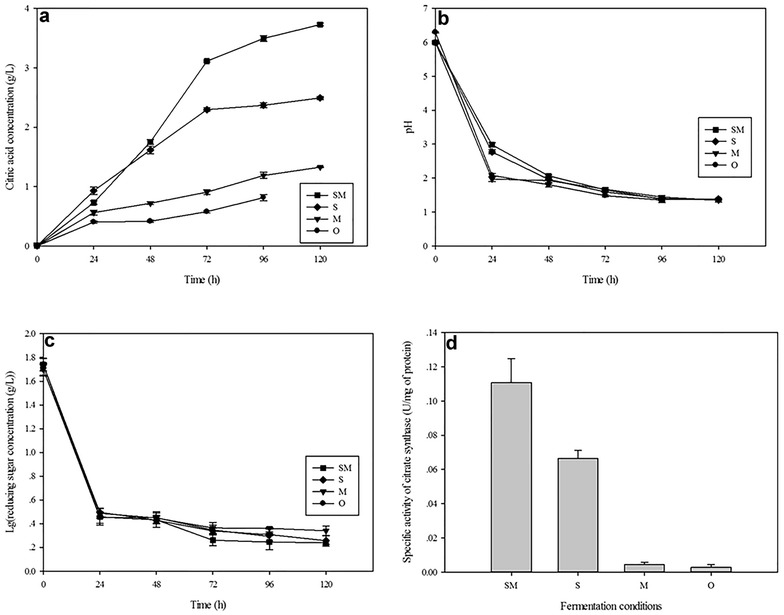
Citric acid accumulation (**a**), pH values change (**b**), reducing sugar consumption (**c**) and citrate synthase activities (**d**) of *A. niger* sp. ZJUY under different cultivation conditions. Citrate synthase activities are the values determined at the end point of fermentation. *SM* SVH and methanol, *S* SVH, *M* methanol, *O* the original conditions

Notably, the addition of SVH and methanol enhanced CA production by *A. niger* using glucose and potato starch as the substrates (Fig. 3a). The optimized conditions produced a CA concentration of 3.729 g/L, which is higher than the CA concentrations of 1.2 mg/g and 0.45 g/L obtained using sugar cane bagasse (Ali and Haq 2005) and apple pomace (Ali et al. 2015), respectively, and close to the theoretically predicted concentration of 3.673 g/L. These results demonstrated the accuracy and applicability of the CCD model as a useful optimization method for biotechnological applications. The final CA concentration produced by this approach was 4.6-fold higher than the control, 0.49-fold higher than that produced in the absence of methanol, and 1.8-fold higher than that produced without SVH, indicating SVH is a cost-effective additive in CA production, and its combination with the permeabilization agent methanol shows an apparent synergistic effect in CA accumulation.

In addition, the pH of the four different cultivation systems behaved similarly with prolonged incubation times (Fig. 3b). All pH values dramatically declined at the initial phase, and then flattened out. It was also noted that pH in systems with SVH was obviously higher than others during the first 48 h, after which the pH curve gradually reduced with that of the others. The influence of pH on CA production has been confirmed by some researchers, and Aravantinos-Zafiris et al. (1994) observed a dramatic increase in CA production when the pH was increased from 3 to 4 and an optimum range fell from pH 4–6. However, CA production can considerably increase the acidity of the fermentation broth, consequently limiting the microorganism’s capacity to ferment sugars. To offset this, Rivas et al. (2008) added CaCO_3_ in the fermentation system to neutralize acidification and obtained positive results. In the present study, pH continuously decreased with increased CA concentration. Finally, the ultimate pH values were stabilized to approximately 1.3. During the fermentation period, SVH neutralized the acidification caused by CA release in the early stages, and maintained system a suitable pH to conduct CA fermentation. Thus, the alleviation of acidification may be one means by which SVH enhances CA accumulation.

Figure 3c showed that SVH could promote decomposition and utilization of the reducing sugar. The reducing sugar residues in the systems with SVH were generally below that observed in the absence of SVH, and methanol accelerated reducing sugar consumption. Citrate synthase (CS) is directly related to CA synthesis, and the fluctuation of citroyl synthetase activities perfectly matched with CA production under different fermentation systems. The strains cultivated in the optimized conditions displayed a highest CS activity (0.11 U/mg), which was somewhat higher than that in the absence of methanol, whereas CS showed the lowest activities in the original conditions (Fig. 3d). This phenomenon indicated SVH could improve CS activity and promote CA production by *A. niger*.

### Analysis of fermentation kinetics

A comparison of kinetic parameters relating to the effects of SVH and methanol added 24, 48, or 72 h after inoculation on CA production by *A. niger* showed significant improvements in *Q*_p_, *Y*_p/s_, and *Y*_x/s_ over those obtained in the system without methanol or SVH. However, when the culture was monitored for *Y*_x/s_, the value of control samples were significantly higher than that of other systems, demonstrating that the control was mostly attributed to vegetative growth during this stage, and accumulated relatively high biomass.  In this research, all treatments with methanol, SVH, or both showed higher *Q*_s_ than that of control, and higher *Q*_s_ value of SM and S systems matched exactly with higher citrate synthase activities and the CA output presented in Table 5.

**Table 5:** Comparison of kinetic parameters for CA fermentation by *A. niger* sp. ZJUY under different fermentation systems

Kinetic parameters	Control	SM			S			M		
		24 h	48 h	72 h	24 h	48 h	72 h	24 h	48 h	72 h
*Q*_p_	0.001244	0.0426	0.0496	0.0384	0.0286	0.0285	0.02	0.006617	0.007201	0.008691
*Y*_p/s_ (g/g)	0.1516	1.7454	0.4612	0.5023	0.3139	0.2879	0.263	0.1194	0.0907	0.1598
*Y*_x/s_ (g cells/g)	10.4660	8.0882	7.8851	8.5778	3.2607	2.6796	1.6608	2.647	4.6332	6.3582
*Q*_s_	0.008421	0.0322	0.108	0.0764	0.0928	0.099	0.0761	0.0565	0.0796	0.0542
LSD	0.5359	1.0517	1.1139	1.6851	1.1123	1.3153	1.1788	0.1975	0.6522	0.4612
Significance level	HS	HS	HS	HS	HS	HS	S	HS	HS	HS

Significant differences were determined by one-way ANOVA followed by LSD post hoc test using software SPSS, version 16.0, (SPSS Inc., Chicago, IL, USA). Differences were considered to be significant at *p* < 0.05

*HS* highly significance, *S* significance*SM* SVH and methanol, *S* SVH, *M* methanol

### SEM and flow cytometry of *A. niger* cells under the different incubation conditions

**Fig. 4:**
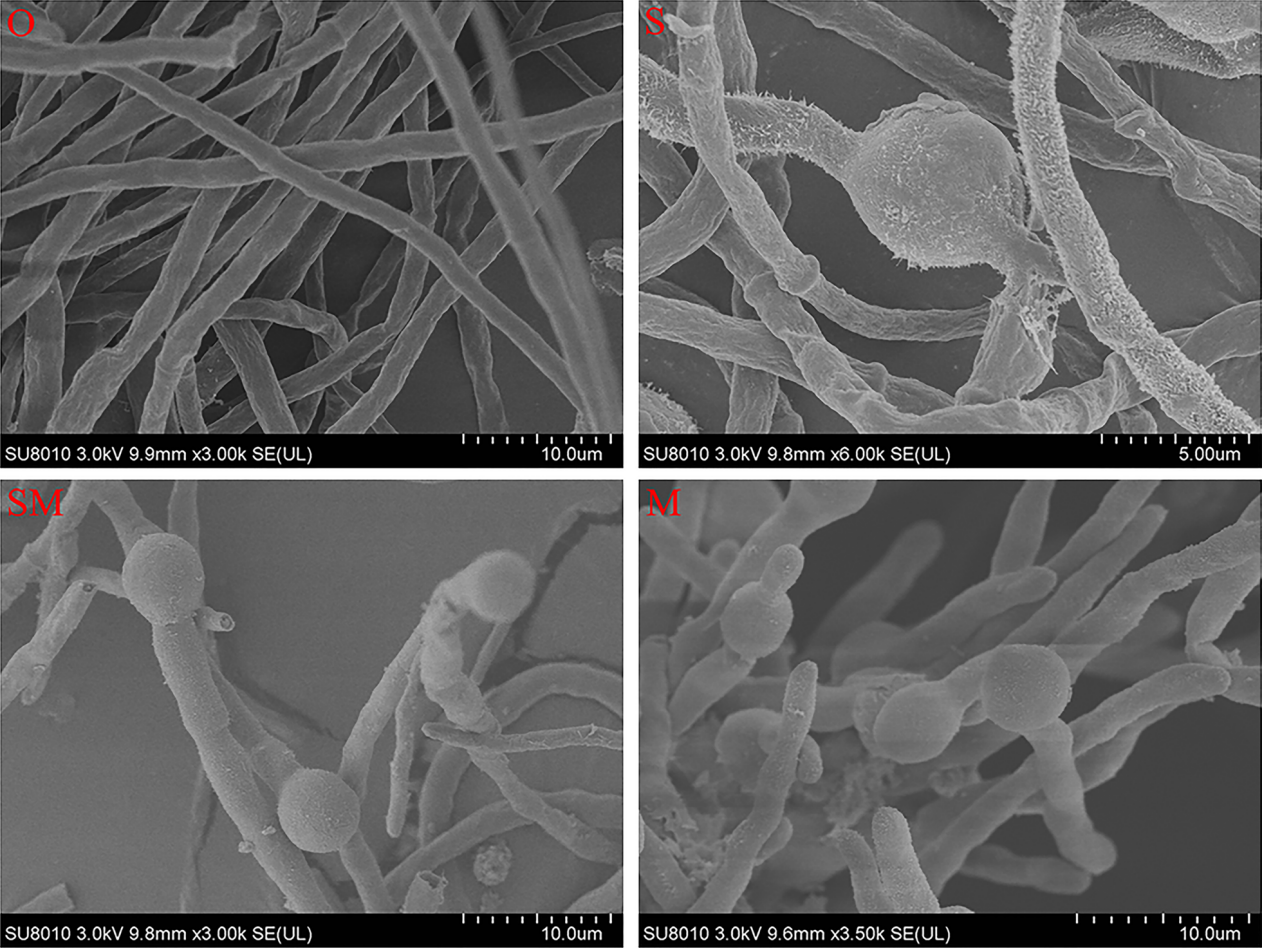
The scanning electron microscope (SEM) graphs of *A. niger* sp. ZJUY cell surface in absence of both SVH and methanol (O), presence of SVH (S), presence of both SVH and methanol (SM), and presence of methanol (M)

**Fig. 5:**
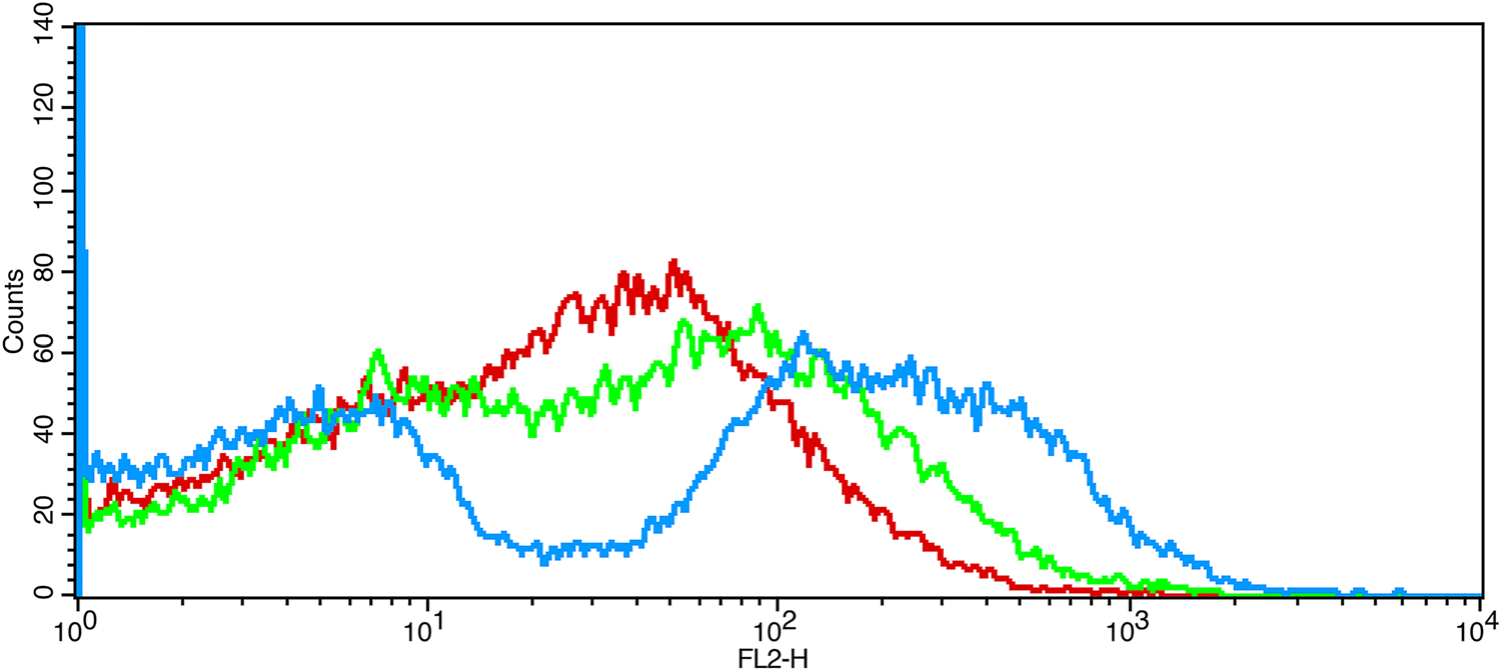
Cell viability was detected by flow cytometry of *A. niger* sp. ZJUY cells, grown on methanol (*blue-broken line*), SVH (*green-broken line*), and methanol supplemented with SVH (*red-broken line*)

Scanning electron microscope of the *A. niger* surface showed that all mycelium produced conidiophores, except for those incubated under the original conditions, in which only spindly mycelium were observed, and methanol stress could promote the formation of conidiophores in the process of fermentation. It is also worth mentioning that the amount of conidiophores was markedly reduced in the system containing SVH, as compared to those where SVH was absent (Fig. 4), suggesting that SVH could relieve the toxic effect of methanol by maintaining *A. niger* in a prolonged vegetative growth phase, and SVH-methanol played a better synergistic effect on CA production. Moreover, *A. niger* cell membrane integrity showed that the death rates of spores in the absence of SVH were slightly higher than other systems during the early phase, and the death rates of spores decreased from 48.21% (absence of SVH) to 33.97% (absence of methanol) to 20.35% (optimized conditions) at the terminal stage. The phenomenon suggested that SVH could effectively maintain membrane integrity. Further, when the fluorescence intensity reached from 10 to 100, the death rate decreased in the beginning and then increased in the absence of SVH (blue-broken line), likely since spores being detected were in different life cycles, wherein methanol promotes apoptosis of spore-bearing and new generated spores. Overall, SVH may prevent the mycelium from the toxic effect of methanol and sustain the cell membrane integrity (Fig. 5).

## Discussion

Bioconversion of the biomass from agro-industrial wastes to produce beneficial fermentation products has received increasing attention in recent years. SVH has been proved to be an effective nutrient supplier for single cell oil production by *Trichosporon fermentans* in previous studies. With SVH as substrate, lipid production was markedly improved compared with that in the original medium (Zhan et al. 2013). Our preliminary experiments indicated that SVH could also be a superior additive for organic acid fermentation by *A. niger*. In the present study, the CA-fermentation conditions were optimized based on RSM, and the positive effect of SVH on CA accumulation was explored. Results showed that a 12% SVH addition could effectively promote CA production and the interaction of initial pH-fermentation temperature, pH-fermentation period, and pH-SVH significantly affected CA productivity (*p* < 0.05). Zhan et al. (2013) have reported that SVH is abundant in organic components and contains a relatively high amount of minerals; the organic components include reducing sugars, some crude polysaccharides, as well as little nitrogen and soluble protein. The considerable concentration (40.83 g/L) of reducing sugars in SVH can be used as a good supplement to glucose during the accumulation of CA. Khare et al. (1995) claimed that the organic nitrogen sources—such as peptone—did not significantly increase CA yield. However, in contrast, Zhang et al. (1999) investigated the relationship between CA output and the amount of organic nitrogen in the fermentation medium, and found that the CA output increased by 27% with the optimum amounts of some amino acid (e.g., Thr, Met) or when enzymatic protein hydrolysate from corn was added to low quality sweet potato powder during fermentation. Our findings were consistent with the latter, in that the amount of total nitrogen in the SVH was 0.94 g/L, indicating that SVH could be an excellent media supplement to offset the absence of certain essential amino acid required for promoting the growth of fungus and activating the citrate synthase system. Thus, the addition of SVH could elevate the CA production to some extent. Besides, SVH afforded some essential trace elements (Ca^2+^, Mg^2+^, Fe^2+^, Mn^2+^, Cu^2+^, etc.) for viable microorganisms. Ali and Haq (2005) found that copper sulfate remarkably enhanced CA production. The distinct nutritional properties of SVH might explain its positive effect on CA accumulation, indicating its great potential for use in the fermentation industry.

Methanol could enhance the CA secretion by altering membrane permeability. Mirbagheri et al. (2011) proposed that the permeabilization process only alters the intracellular lipid bilayer, while leaving the outer membrane intact. However, methanol still posed as a toxic threat to cell growth to some extent. Our SEM analyses showed that the largest number of conidiospores distributed above the mycelium in systems containing only methanol. Moreover, flow cytometry of spores under different fermentation conditions showed a decreased viability as compared to others during the early and late phase, which indicated that methanol had toxic effects on living cells and the presence of methanol accelerated *A. niger* reproductive growth.

This study verified the speculative synergistic effect of methanol and SVH as enhancers in CA production. The optimal methanol-SVH fermentation system resulted in a maximum citric acid concentration of 3.729 g/L, increased by 3.6-fold over the original condition, 0.49-fold over the optimized conditions without methanol, and 1.8-fold over the optimized conditions in the absence of SVH. The mechanisms of how methanol and SVH cooperatively increased CA production were initially explored from the perspective of fermentation dynamics, morphology, and cell viability. Fermentation kinetic studies showed that all the values for the SVH-methanol system were significantly improved with respect to *Q*_p_, *Y*_p/s_, and *Y*_x/s_ over those obtained in systems without methanol or SVH. This improvement confirmed that the combination of SVH and methanol increased uptake of the carbon source and its subsequent assimilation. However, it was noted that when the culture was monitored for *Y*_x/s_, the value of controls were significantly higher than that of other systems, which demonstrated the control mainly conducted vegetative growth during this stage, produced much biomass but accumulated little CA. Higher *Q*_*s*_ values indicate a higher biomass conversion and productivity. In our study, all treatments added with methanol, SVH, or both showed higher *Q*_*s*_ than controls, and the higher *Q*_*s*_ value of the SM and S systems perfectly matched with higher citrate synthase activities and CA accumulation.

Morphology and cell viability studies showed that SVH could effectively relieve methanol toxic effect on growth of *A. niger* and maintain high CA productivity. In the SVH-added system, the death rate of *A. niger* cells was fairly low and only few conidiospores were observed. These results might illustrate the remarkable synergistic effect of two enhancers on CA production except for nutritional factor.

In our preliminary experiment, we employed SVH as a sole carbon source in mineral medium with starch and glucose as control. However, CA production was relatively lower than the otherwise, and when SVH was added as a nutrient supplement instead of the same amount of carbon source, CA production was considerably improved. The results suggested that SVH acted as a beneficial additive rather than alternative carbon sources in our study, maybe due to its poor carbon source and low C/N. A similar phenomenon has also been found by Shen et al. (2015), who showed that the co-fermentation of molasses and SVH increased the lipid output by 35% when 10% SVH was added, compared to the poor lipid accumulation on pure molasses by *T. fermentans*.

The CA yield of this work was significantly lower when compared with those of many other reports. For example, Roukas achieved a maximum CA concentration of 85.5 g/L from carob pod extract in surface fermentation (Roukas 1998), Hang obtained a high CA concentration of 100 g/kg in solid-state fermentation with kiwi fruit peel as only carbon source (Hang and Woodams 1998), then the successful cases were also repeated by Angumeenal (73 g/L), Dhillon (44.9 g/kg), and Vandenberghe (88.1 g/kg) (Angumeenal and Venkappayya 2005; Dhillon et al. 2011; Vandenberghe et al. 2000). However, the weak CA production in our study was mostly attributed to differences in the fermenting strain. The main purpose of this study was to explore a new way for the resource utilization of sweet potato vine waste. Certainly, application of industrial strains in CA fermentation could be more meaningful, while the fact that SVH can be used as a beneficial additive to enhance CA production of a weak production strain actually indicated its broad applicability in the fermentation industry.

## Conclusions

The main purpose of this study was not to produce citric acid in amount but to explore a new way for the resource utilization of agricultural waste-sweet potato vine. What’s more is, we found that the combination of methanol and SVH displayed a significant synergistic effect on CA accumulation even with a weaker strain, which suggested its great application potential in fermentation.
